# RSK Activation of Translation Factor eIF4B Drives Abnormal Increases of Laminin γ2 and MYC Protein during Neoplastic Progression to Squamous Cell Carcinoma

**DOI:** 10.1371/journal.pone.0078979

**Published:** 2013-10-28

**Authors:** Martin Degen, Patricia Barron, Easwar Natarajan, Hans R. Widlund, James G. Rheinwald

**Affiliations:** 1 Department of Dermatology, Brigham and Women's Hospital and Harvard Skin Disease Research Center, Boston, Massachusetts, United States of America; 2 Section of Oral and Maxillofacial Pathology, University of Connecticut Health Center, Farmington, Connecticut, United States of America; Aix-Marseille University, France

## Abstract

Overexpression of the basement membrane protein Laminin γ2 (Lamγ2) is a feature of many epidermal and oral dysplasias and all invasive squamous cell carcinomas (SCCs). This abnormality has potential value as an immunohistochemical biomarker of premalignancy but its mechanism has remained unknown. We recently reported that Lamγ2 overexpression in culture is the result of deregulated translation controls and depends on the MAPK-RSK signaling cascade. Here we identify eIF4B as the RSK downstream effector responsible for elevated Lamγ2 as well as MYC protein in neoplastic epithelial cells. Premalignant dysplastic keratinocytes, SCC cells, and keratinocytes expressing the E6 oncoprotein of human papillomavirus (HPV) type 16 displayed MAPK-RSK and mTOR-S6K1 activation and overexpressed Lamγ2 and MYC in culture. Immunohistochemical staining of oral dysplasias and SCCs for distinct, RSK- and S6K1-specific S6 phosphorylation events revealed that their respective upstream pathways become hyperactive at the same time during neoplastic progression. However, pharmacologic kinase inhibitor studies in culture revealed that Lamγ2 and MYC overexpression depends on MAPK-RSK activity, independent of PI3K-mTOR-S6K1. eIF4B knockdown reduced Lamγ2 and MYC protein expression, consistent with the known requirement for eIF4B to translate mRNAs with long, complex 5′ untranslated regions (5′-UTRs). Accordingly, expression of a luciferase reporter construct preceded by the Lamγ2 5′-UTR proved to be RSK-dependent and mTOR-independent. These results demonstrate that RSK activation of eIF4B is causally linked to elevated Lamγ2 and MYC protein levels during neoplastic progression to invasive SCC. These findings have potential clinical significance for identifying premalignant lesions and for developing targeted drugs to treat SCC.

## Introduction

Squamous cell carcinoma (SCC) is an aggressive cancer that arises from stratified epithelia, including the epidermis and the bronchial, cervical, and oral epithelia. Oral SCC is a significant health problem, with 27,000 new diagnoses and 5,500 deaths per year in the U.S. alone [1]. Genetic alterations frequently present in advanced oral and oropharyngeal SCCs include mutations of *TP53*, *CDKN2A*, *HRAS*, *PTEN*, and *PIK3CA* and gene amplification of *EGFR* and *MYC* [2], [3]. Many of these alterations result in activation of the MAPK pathway (i.e., RAS-RAF-MEK-RSK) or the PI3K-AKT-mTOR pathway, or both [2]–[5]. The major carcinogenic factors contributing to acquisition of these genetic changes are tobacco mutagens. However, 6% of oral and a higher proportion of oropharyngeal SCCs involve infection with high-risk HPV types 16 or 18 and integration and expression of their E6 and E7 viral oncogenes [6]–[8], the activities of which can replace mutations in some of the genes listed above to confer neoplastic properties.

Deeply invasive or regionally metastatic oral SCC cannot be surgically resected and is refractory to cure by conventional chemotherapy, targeted kinase inhibitor, or blocking antibody approaches [9]–[11]. Identification of early, premalignant stages that are completely resectable should greatly increase cure rate. Oral SCCs usually develop from visually identifiable, dysplastic precursor lesions. Such dysplasias have an incidence rate of ∼1–3% in adults [12]; however the majority never progress to invasive SCC and current conventional histopathologic criteria cannot predict which lesions will progress [12]–[15]. Thus, there is an urgent need both to identify predictive biomarkers for evaluating dysplasias and to better understand the mechanisms of neoplastic progression in order to reveal potential drug targets for therapy.

Abnormal overexpression of the Lamγ2 subunit of the stratified epithelial basement membrane protein Laminin-332 is a characteristic of many types of invasive carcinoma [16]–[18] and is already present in many preinvasive dysplasias of the oral epithelium and epidermis [19], [20]. We recently described a culture system that recapitulates the absence of Lamγ2 expression by normal epithelium and the abnormal Lamγ2 overexpression by premalignant dysplasias and SCCs *in vivo* [21]. In this system, normal human keratinocytes cease Lamγ2 synthesis as they become confluent whereas premalignant keratinocytes, SCC cells, and keratinocytes expressing the HPV16 E6 oncoprotein continue synthesizing Lamγ2. EGFR/MAPK/RSK hyperactivity, detectable in culture and *in vivo* by RSK-specific phosphorylation of ribosomal protein S6 at its S235 residue, proved to be essential for driving Lamγ2 overexpression. Additionally, Lamγ2 overexpression correlated closely with RSK-mediated phosphorylation of the translation regulatory factor eIF4B [21]. Activated eIF4B is a cofactor for eIF4A, an RNA helicase that is required to unwind the long, stem-looped 5′-UTRs of certain mRNAs, such as those of MYC, ODC, and BCL2, to expose the AUG translation initiation codon [22]–[26].

Here we characterize the molecular basis of Lamγ2 overexpression and of the MYC overexpression that invariably accompanies it in SCC cells, premalignant keratinocytes, and keratinocytes expressing the HPV16 E6 viral oncoprotein. Using specific antibodies that detect and distinguish MAPK/RSK from mTOR/S6K1-dependent phosphorylation events on S6, we find concurrent hyperactivation of both pathways during epithelial neoplastic progression *in vivo*, presaging Lamγ2 overexpression. Using genetic and pharmacologic inhibitor approaches in culture, we find that RSK activation of eIF4B, independent of PI3K/mTOR, is critical for Lamγ2 protein expression and that the Lamγ2 mRNA 5′-UTR sequence is responsible for this regulatory mechanism.

## Materials and Methods

### Cells and Cell Culture

Human cell lines used in this study were the premalignant dysplastic oral keratinocyte line POE9n [27], [28]; the epidermal SCC cell line SCC-13 [29] and the oral SCC cell line SCC-68 [21]; the normal epidermal keratinocyte primary line Strain N [29], [30]; and N/E6(JH26) [21], a derivative of strain N engineered by retroviral transduction to express the JH26 mutant form of the E6 oncoprotein of HPV16 [31], [32]. All of these cell lines were derived in the Rheinwald lab and cultures for experiments were initiated from archival frozen stocks in the Rheinwald lab's collection. Their derivation and use in this study were approved by the Brigham and Women's Hospital's Human Studies IRB.

Cells were cultured in keratinocyte serum-free medium (Ksfm) (GIBCO/Life Technologies, Carlsbad, CA), +25 µg/ml bovine pituitary extract +0.2 ng/ml epidermal growth factor (EGF) +0.4 mM CaCl_2_ +penicillin/streptomycin (pen/strep) (GIBCO/Life Technologies) [27]. To keep cells healthy and metabolically active at high density, after reaching ∼40% confluence cultures were fed daily with “1∶1 medium” (1∶1 (vol∶vol) Ca^2+^-free DMEM (GIBCO/Life Technologies): Ksfm +25 µg/ml bovine pituitary extract +0.2 ng/ml epidermal growth factor (EGF) +0.1 mM CaCl_2_, +pen/strep, as described [21]. This lower calcium medium prevented stratification and accumulation of suprabasal, terminally differentiated cells, the percentages of which could vary among cell lines and treatment conditions. Thus, in this medium only the relevant basal cell populations of cell lines and treatment conditions were analyzed.

HEK293T cells [33] (Broad Institute, Cambridge, MA), used for lentivirus production, were grown in DMEM +10% newborn bovine calf serum (Hyclone/Thermo Scientific, Rockford, IL) + pen/strep.

### Small molecule kinase inhibitors

The EGFR inhibitor gefitinib [34], [35] (provided by Pasi Janne, Dana-Farber Cancer Institute, Boston, MA) was used at 1 µM, the RSK inhibitor BI-D1870 [36] (Symanis Limited, Auckland, NZ) at 2.5 µM, the MEK inhibitor U0126 [37] (Cell Signaling Technology) at 5 µM, the mTORC1/2 inhibitor Ku-0063794 [38], [39] (Chemdea, Ridgewood, NJ) at 500 nM, the mTORC1 inhibitor rapamycin (Cell Signaling Technology) at 10 nM, and the PI3K p110α subunit inhibitor PIK-75 [40] (Selleckchem, Houston, TX) at 40 nM. Inhibitors were added to the medium from 1,000X concentrated solutions in DMSO, stored frozen between uses.

### Antibodies

Murine monoclonal antibodies used were: Laminin γ2 (clone D4B5 [41], Chemicon, Billerica MA), Laminin β3 (clone 17, BD Transduction Laboratories, Franklin Lakes, NJ). Rabbit monoclonal antibodies used were: MYC (cloneY69, Epitomics, Burlingame, CA), and phospho- (p-)S6(S235/236) (clone 91B2), p-S6(S240/244) (clone D68F8), p-AKT(S473), all from Cell Signaling Technology (Danvers, MA). Rabbit polyclonal antibodies used were: β-actin (A-2066, SIGMA-Aldrich); p-eIF4B(S442) (AbCam, Cambridge, MA), p-RSK(S380) (clone AF889) (R&D Systems, Minneapolis, MN); and ERK1/2, p-ERK1/2(T202/Y204), and eIF4B (all from Cell Signaling Technology).

### Western blot analysis

Cultures were lysed in reducing Laemmli SDS sample buffer, separated by SDS-PAGE on 4–20% gradient gels (Bio-Rad, Hercules, CA), blotted to polyvinyldifluoride membranes (Bio-Rad), incubated primary and with peroxidase-conjugated antibodies, treated with chemiluminescence reagent, and exposed to HyBlot CL film (Denville Scientific, Metuchen, NJ), as described [21]. Blots were analyzed densitometrically using ImageJ software version 1.45 (NIH, Bethesda, MD; http://rsbweb.nih.gov/ij). Two different film exposures of each blot were analyzed. Band densities of each sample were normalized to the density of the actin band of the same sample and compared to the densities of control lanes in the same film exposure.

### Tissue samples and immunohistochemistry

Ten formalin-fixed, paraffin-embedded, lateral tongue tissue specimens from the U. Conn. Oral Pathology archival collection used in this study were obtained with written informed consent for their use in research by the donors and analyzed anonymously with the approval of the Brigham and Women's Hospital Human Studies IRB. H&E stained slides of these specimens were evaluated by one of us (EN) who is an oral pathologist and included normal epithelium, dysplasias, and invasive SCC. Sequential 5 µm paraffin sections (not baked at 50°C after sectioning to preserve the Lamγ2 antigen [19]) were immunostained by the avidin/biotin/peroxidase complex method and Vector Red as the color reagent (Vectastain Elite ABC kit, Vector Laboratories, Burlingame, CA), examined using a NIKON Eclipse TE2000-S microscope, and photographed with a SPOT Insight QE camera, as described [21]. Immunostained slides were quantified for% basal layer p-S6(S235), p-S6(S240), and Lamγ2 as follows: the total length in section of the dysplastic area of each specimen was measured in the H&E-stained slide and the percentage of the dysplastic region with p-S6 positive and Lamγ2 positive basal cells was determined.

### Bicistronic luciferase constructs and reporter assays

The pDL-N bicistronic Renilla/Firefly luciferase reporter plasmid [42] was used for translation reporter assays. Translation initiation of the Renilla luciferase cistron is 5′ cap-dependent, and translation initiation of the Firefly luciferase cistron is independent of 5′ cap- or other eIF4-dependent mechanisms because it is preceded by the hepatitis C virus internal ribosomal entry site (IRES). The 5′-UTRs of the *LAMC2* gene (encoding Lam2) and of the *ODC* gene (encoding ornithine decarboxylase) were cloned into pDL-N upstream of the Renilla AUG translation initiation site. The pDL-N/(ODC 5′UTR) plasmid was provided by Drs. Nina Ilic and Tom Roberts, Dana-Farber Cancer Institute. We generated the pDL-N/(Lamγ2 5′UTR) plasmid by PCR-amplifying the 314 bp Lamγ2 5′UTR sequence from genomic DNA isolated from cultured human keratinocytes using the primer pairs: FWD: AGAAGCTTCTTGGCCCGGGCCAGGTGTGC, and REV: AGAAGCTTGGCGGGGCCGGGCCGCTCAGT, containing HindIII sites (underlined above), and cloning into the HindIII site upstream of the Renilla translation initiation site in pDN-L.

SCC-13 cells were transfected in 24-well plates in quadruplicate using 50 ng of the luciferase reporter plasmids and linear polyethylenimine 25kDa (PEI-25K; Polysciences, Inc., Warrington, PA) as transfection reagent. Cells were lysed 2 d post-transfection in 100 µl passive lysis buffer (dual luciferase reporter kit, Promega, Madison, WI) per well. Extracts were assayed using a dual luciferase system (Promega) and measured in a GLOMAX 96 microplate luminometer (Promega). When used, kinase inhibitors were added 1 d after transfection and the cells lysed 1d thereafter. Fold-change in activity was calculated as the ratio of Renilla and Firefly luciferase activities in pDL-N/(5′-UTR) construct-transfected cells compared to the ratio in pDL-N-transfected cells. Standard error of the mean was calculated from results of four experiments, each performed in quadruplicate. Student's t test was calculated to determine statistical significance of differences.

### Quantitative PCR

Total RNA was isolated from cells using the RNeasy Plus Mini Kit (Qiagen, Valencia, CA) and cDNA synthesized from 500 ng total RNA using the iScript cDNA synthesis kit (Bio-Rad). mRNA levels were measured by quantitative real-time PCR (qPCR) using FAST SYBR-Green PCR Master Mix (Applied Biosystems, Foster City, CA) on an ABI StepONE Plus Instrument (40 cycles of 95°C for 15 s and 58°C for 30 s). Amplicons used were: Laminin γ2 (gene name: *LAMC2*) (FWD: 5-CTCTGCTTCTCGCTCCTCC-3′, and REV: 5′-TCTGTGAAGTTCCCGATCAA-3′); MYC (*MYC*) (FWD: 5′-TTTCGGGTAGTGGAAAACCA-3′, and REV: 5′-CACCGAGTCGTAGTCGAGGT-3′); eIF4B (*EIF4B*) (FWD: 5′-TTTCCCTCTCCCAACATGG-3′, and REV: 5′-GTGCTTCCTCCACCAGTACC-3′); GAPDH (*GAPDH*) (FWD: 5′-GAGCCTCAAGATCATCAGCA-3′, and REV: 5′-ACAGTCTTCTGGGTGGCAGT-3′). Relative expression was calculated using the ΔΔC_t_ method, normalizing values to GAPDH within each sample, and calculating standard error of the mean from the results of triplicate aliquots.

### Lentiviral shRNA and expression vectors and transduction

Three short hairpin sequences designed to target specifically mRNAs encoding human eIF4B (shEIF4B) and a control hairpin targeting Luciferase (shLUC) in the pLKO.1 lentiviral backbone were obtained from the RNAi Consortium (TRC Broad Institute, Cambridge, MA). The eIF4B shRNA vectors targeted the following sequences in eIF4B mRNA:

- Clone #1: 5′-GCGGAGAAACACCTTGATCTT-3′,
- Clone #3: 5′-CCAACTTCTAAACCTCCCAAA-3′,
- Clone #4: 5′-CTACCCTATGATGTTACAGAA-3′.

To produce lentiviral supernatants, HEK293T cells in 6-wells were transfected with 1 µg of shRNA vector plasmid, 1 µg pCMV-GagPol(psPAX2)Δ8.9, and 100 ng pCMV-VSVG, using PEI-25K as transfection reagent. Lentiviral supernatants were collected in 1∶1 medium 48 h and 72 h after transfection, passed through 0.45 µm pore filters, and stored at −80°C before use. Cells plated 1 d previously at 10^5^ cells/9 cm^2^ well in Ksfm were transduced for 6–7 h with lentiviral supernatants containing 2 µg/ml polybrene (SIGMA) as described [30]. Transduced cells were subcultured the next day into Ksfm +1 µg/ml puromycin and selected for 3 d to obtain pure transductant populations.

## Results

### Lamγ2 and MYC overexpression and eIF4B phosphorylation are EGFR/MAPK-dependent but PI3K/mTOR/S6K1 -independent

**Figure 1:**
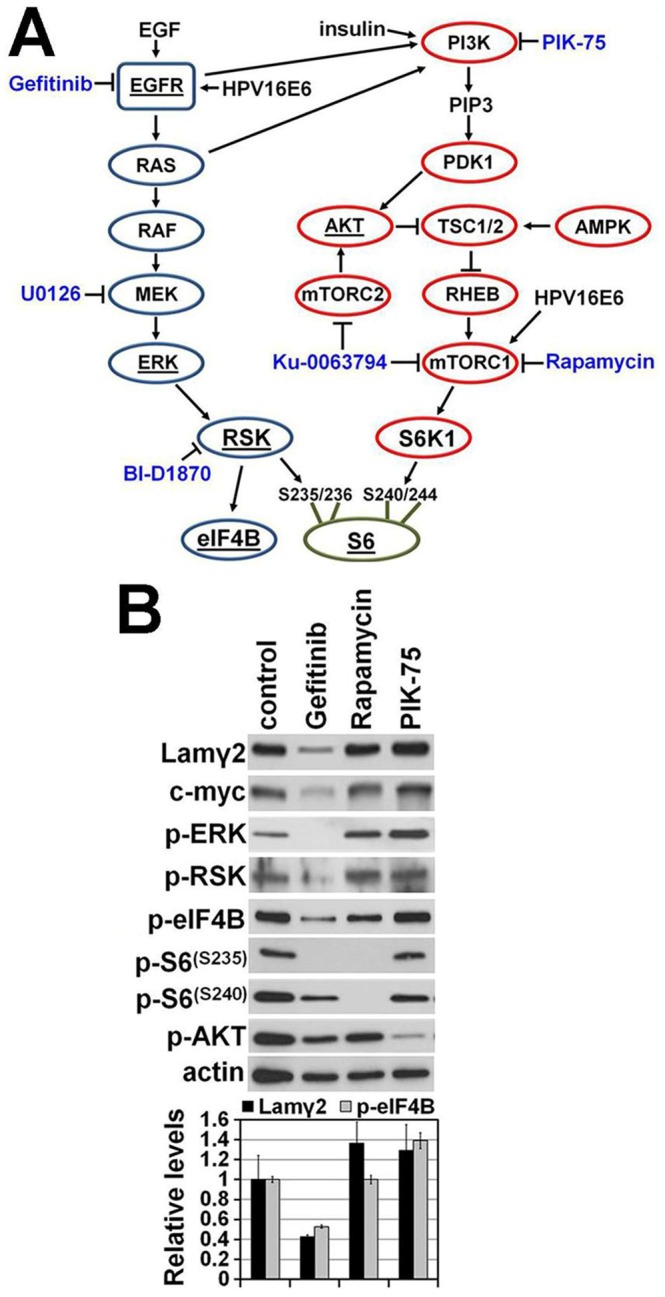
Effects of inhibiting MAPK vs. PI3K/mTOR pathway kinases on Lamγ2 and MYC expression. **A**) Elements of the EGFR/RAS/RAF/MEK/RSK (blue) and PI3K/AKT/mTOR (red) kinase cascade signaling pathways and their downstream targets eIF4B and S6. Arrows indicate that the upstream protein activates the downstream target, whereas lines terminating in a crossbar indicate that the upstream protein inhibits the downstream target. Kinase inhibitors are in blue font. Proteins examined for their phosphorylation states by Western blotting are underlined. **B**) Western blot of confluent cultures of SCC-68 treated for 24 hr with the EGFR kinase inhibitor gefitinib, the mTORC1 inhibitor rapamycin, or the PI3 kinase p110α-specific inhibitor PIK-75. Densitometric analysis of the Lamγ2 (black bars) and p-eIF4B (gray bars) bands of each lane is shown in the bar graph below the blot. Lamγ2 and p-eIF4B levels were normalized to the actin level in each lane and then levels of Lamγ2 and p-eIF4B in inhibitor-treated cultures were expressed relative to those in the untreated control culture.

To extend our previous finding that Lamγ2 and MYC overexpression correlated with MAPK pathway hyperactivity in SCC cells [21], we investigated more thoroughly the possibility of a role for the PI3K/mTOR pathway. To this end, we examined the effects on Lamγ2 and MYC expression of PIK-75, an inhibitor of the PI3K subunit p110α [40], and of rapamycin, a specific inhibitor of mTORC1, comparing these to gefitinib, an inhibitor of EGFR kinase that can stimulate both the MAPK and PI3K signaling cascades (Fig. 1A).

As predicted [21], gefitinib reduced Lamγ2 and MYC expression, associated with reduced levels of p-ERK, p-RSK, p-S6(S235), and p-eIF4B (Fig. 1B). Neither PIK-75 nor rapamycin reduced Lamγ2 and MYC expression and p-ERK, p-RSK, and p-eIF4B levels were unaltered by these inhibitors. The activities of PIK-75 and rapamycin in this system were demonstrated by the reduction of p-AKT levels by PIK-75 and of p-S6(S240) levels by rapamycin. Notably, PIK-75 did not affect p-S6(S240) levels (Fig. 1B). Considering that our culture conditions do not impose nutrient or growth factor limitations which otherwise might instigate AMPK activation of TSC1/2 (Fig. 1A), it is not unexpected that cells were independent of PI3K/AKT-dependent activation of mTORC1 and its downstream target S6K1.

The great reduction of p-S6(S235) levels by gefitinib and of p-S6(S240) by rapamycin (Fig. 1B) support the conclusion that these sites are phosphorylated by RSK and S6K1, respectively. On the other hand, rapamycin inhibition of mTORC1 and consequently inactive S6K1 also markedly reduced p-S6(S235) levels, despite RSK remaining active as evidenced by the maintenance of p-eIF4B levels in the presence of rapamycin (Fig. 1B). These results are consistent with the conclusion that phosphorylation of S6(S240) by S6K1 is an essential prerequisite for subsequent phosphorylation of S6(S235) by RSK.

### *In vivo* phosphorylation status of S6 as immunohistochemical markers of MAPK and mTOR activation

Our recent studies identified regions in oral epithelial and vulvar epidermal SCCs and dysplasias that contained immunohistochemically-detectable p-S6(S235) in cells of the basal layer [21], [43]. The RSK- and S6K1-specific phosphorylations of S6 we found by Western blotting in cultured SCC cells (Fig. 1B) provided the rationale for using p-S6(S235)- and p-S6(S240)-specific antibodies on pathology tissue sections to detect activation of (MAPK/RSK + mTORC1/S6K1) vs. activation of mTORC1/S6K1 alone. We asked whether an event causing mTORC1/S6K1 activation precedes an event causing MAPK/RSK activation during epithelial neoplastic progression. If so, this would be manifested as an immunostaining pattern in which p-S6(S235) positive cells always lie within a larger field of p-S6(S240) positive cells.

**Figure 2:**
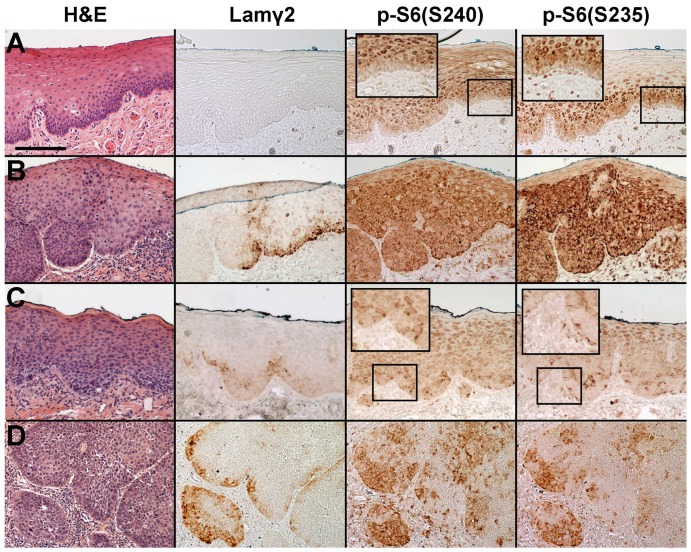
Coincidence of p-S6(S235) and p-S6(S240) detectable in human oral dysplastic lesions and SCCs *in vivo*. Sections of normal (**A**) and dysplastic (**B-C**) epithelium, and invasive SCC (**D**), stained with H&E and immunostained for Lamγ2, p-S6(S240) and p-S6(S235). Scale bar: 200 µm. Enlarged insets of some regions are shown for easier viewing of p-S6 staining patterns. Panel A shows a region of Case 2, panel B of case 8, panel C of case 7, and panel D of case 4 as described in Table 1. Note that normal epithelium did not express Lamγ2 and neither S6 phosphorylation event was detectable in the basal cells. Cells in dysplasias and SCCs always showed coincidence of the two S6 phosphorylation events. Dysplasias varied with respect to frequency and intensity of Lamγ2 expression and S6 phosphorylation, with Lamγ2 cells representing a subset of p-S6 positive basal cells and invasive SCCs contained many Lamγ2 and basal layer p-S6 positive regions.

To answer this question, we immunostained 10 human oral dysplasia specimens, some of which also contained regions of normal epithelium and SCC. Normal lateral tongue epithelium present in these specimens always was Lamγ2 negative and p-S6(S240) and p-S6(S235) were confined to the suprabasal cell layers (Fig. 2A). In areas of dysplasia, basal layer p-S6(S240) and p-S6(S235) immunostaining always corresponded exactly, as discussed below. Proportions of the dysplastic regions positive for basal layer p-S6 and Lamγ2 ranged from 0–100% and 0–83%, respectively/ When Lamγ2 was detected in dysplasias and SCCs, it was confined to cells within p-S6(S235) and p-S6(240) positive regions, although not all p-S6 positive cells were Lamγ2 positive (Fig. 2B and Table 1), consistent with our previous studies [21], [43]. (Note that two MYC antibodies that worked in Western blotting and in immunofluorescence of methanol-fixed cultured cells failed to detect this protein in tissue sections, precluding analysis of this protein.)

**Table 1:** Human oral epithelial lesions examined for increased p-S6 and Laminin γ2 by immunohistochemical staining.

Case #	Histopathologic description	% p-S6(S235)+ and p-S6(S240)+	% Lamγ2+	Associated SCC?
1	dysplasia	23	10	yes
2	dysplasia	71	6	no
3	dysplasia	90	37	no
4	severe dysplasia	60	n.d.	yes
5	severe dysplasia	44	33	yes
6	severe dysplasia	69	14	no
7	dysplasia	100	67	yes
8	slight atypia	0	0	no
9	severe dysplasia	34	12	no
10	severe dysplasia	83	83	no

Formalin-fixed, paraffin-embedded specimens of oral lesions were immunostained for p-S6(S235), p-S6(S240), and Lamγ2 and the percentage of the total dysplasia positive in the basal cell layer for these markers determined as described in Methods. Basal layer p-S6(235) and p-S6(240) immunostaining always corresponded precisely. n.d.: not determined.

The immunostaining intensity (and therefore the apparent level of S6 phosphorylation) as well as the proportion of cells detectably positive for p-S6 and Lamγ2 varied among dysplasias (e.g., Fig. 2B,C) (Table 1). Some variability among specimens with respect to immunostaining intensity and continuity could result from different lengths of time before biopsy fixation or of time in fixative before paraffin embedding. Intracellular p-S6 levels may fluctuate more rapidly than that of Lamγ2 protein, the latter which requires 12 hr or more to pass through the endoplasmic reticulum and Golgi and then vacate cells by secretion [21]. Our results are consistent with the conclusion that the degree of MAPK/RSK activation needed to stimulate Lamγ2 overexpression is greater than that required to produce immunologically detectable levels of p-S6(S235).

As expected from our cell culture finding that phosphorylation of S6 at its S240 is a prerequisite for the p-S6(S235) event, regions of dysplasias and SCCs that were basal layer p-S6(S235) positive also were positive for p-S6(S240) (Fig. 2B-D). Our cell culture studies (see Fig. 1B and below) disclosed that both MAPK and mTOR pathways were hyperactive in SCC cells and premalignant keratinocytes. We found no regions of dysplasias or SCCs that were basal layer p-S6(S240) positive but p-S6(235) negative. Thus, we did not detect an early stage of progression in which an event activating the mTOR pathway precedes a separate event activating the MAPK pathway. We concluded from these results that a single event during neoplastic progression activates both an element of the MAPK pathway upstream of RSK and an element of the mTORC1 pathway upstream of S6K1.

### Activation of eIF4B and Lamγ2 mRNA translation are both MAPK/RSK-dependent but mTOR/S6K1-independent

**Figure 3:**
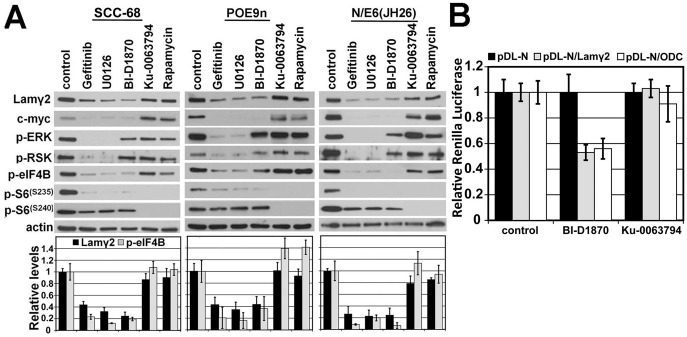
MAPK/RSK-dependent, mTOR/S6K1-independent activation of eIF4B and Lamγ2 mRNA translation. **A**) Western blot analysis of confluent cultures of SCC-68, the premalignant oral keratinocyte line POE9n, and normal primary keratinocyte strain N engineered to express the JH26 mutant of HPV16 E6 (N/E6(JH26). Cultures were treated for 24 hr with the indicated kinase inhibitors and then analyzed for levels of Lamγ2 and MYC protein and for the phosphorylated, activated forms of signaling proteins and translation factors. The Lamγ2 band shown is the 155 kD intracellular form and not the 105 kD form that predominates after secretion and proteolytic processing. The bar graphs below show densitometric analysis of Lamγ2 and p-EIF4B levels in each drug treatment condition relative to untreated control cultures of each line, as described in panel B. **B**) SCC-13 cells transfected with the reporter constructs pDL-N, pDL-N/(Lamγ2 5′-UTR), and pDL-N/(ODC 5-′UTR) with or without the RSK inhibitor BI-D1870 or the mTORC1/2 inhibitor Ku-0063794 and analyzed for Renilla and Firefly luciferase activity. Reduction caused by BI-D1780 in Lamγ2 5′UTR- and ODC 5′UTR-dependent expression had P values for significance of 0.0043 and 0.01, respectively.

We next examined SCC cells and premalignant dysplasia-derived keratinocytes more closely for their MAPK and mTOR signaling pathway activity and dependence on these pathways for Lamγ2 and MYC protein overexpression. We compared confluent cultures of SCC-68 and of the premalignant oral keratinocyte line POE9n. Untreated cultures were compared with replicate cultures treated for 24 hr with inhibitors blocking various kinases in the signal pathways described in Fig. 1A. Western blot analysis (Fig. 3A) disclosed that both the MEK inhibitor U0126 and the RSK inhibitor BI-D1870 reduced p-S6(S235), p-eIF4B, and Lamγ2 and MYC levels. U0126 reduced p-ERK levels but BI-D1870 did not, demonstrating their target specificities. P-eIF4B levels always correlated closely with Lamγ2 and MYC levels, in agreement with our previous study [21]. The mTORC1 inhibitor rapamycin and the mTORC1/mTORC2 inhibitor Ku-0063794 greatly reduced p-S6(S240) and p-S6(S235) levels but had essentially no effect on levels of p-ERK, p-RSK, p-eIF4B, or of Lamγ2 and MYC. Importantly, the rapamycin result confirmed that eIF4B phosphorylation is accomplished by RSK without requiring mTORC1-dependent S6K1 activity.

The oral SCC and dysplasia specimens and the SCC-68 and POE9n cell lines we analyzed above were non HPV-related. However, we have reported recently that high-risk HPV-related vulvar epidermal dysplasias and SCCs contain regions positive for basal layer p-S6(S235) and Lamγ2 [43]. Our earlier study found that an activity of the E6 oncoprotein of HPV16 separate from its ability to target p53 for degradation (disclosed by the E6 mutant E6(JH26)) can instigate Lamγ2 overexpression in keratinocytes [21]. That study followed an earlier one by others reporting that E6 activates mTOR in keratinocytes [44]. We therefore examined confluent cultures of normal primary keratinocytes stably engineered to express E6(JH26) for the activation status of mediators of the EGFR/MAPK and mTOR pathways and for effects of kinase inhibitors on Lamγ2 and MYC expression. Both gefitinib and U0126 reduced Lamγ2 and MYC expression in N/E6(JH26) cells, associated with greatly reduced p-ERK, p-RSK, p-S6(S235), and p-eIF4B levels (Fig. 3A). BI-D1870 reduced p-eIF4B levels and Lamγ2 and MYC expression. As was the case for SCC-68 and POE9n, the mTOR inhibitors Ku-0063794 and rapamycin had little or no effect on Lamγ2 or MYC expression or on p-ERK, p-RSK, or p-eIF4B levels, while blocking the S6K1-dependent S6(S240) phosphorylation and the S6(S235) phosphorylation (Fig. 3A), the latter requiring prior S240 phosphorylation. These results indicate that MAPK and mTOR signaling are hyperactive in non HPV-related SCC and premalignant dysplastic cells and that the E6 viral oncoprotein also activates these pathways, independent of its p53 targeting function. Furthermore, in all cases RSK activity is required for eIF4B phosphorylation and correlates with Lamγ2 and MYC overexpression.

If eIF4B activation is required for Lamγ2 overexpression, then the 5′-UTR sequence of Lamγ2 mRNA should confer dependence for translation on the eIF4B activator RSK. To determine this, we modified the bicistronic Renilla and Firefly luciferase reporter plasmid pDL-N to insert the 5′-UTR of Lamγ2 upstream of the Renilla luciferase coding sequence. We tested this reporter in SCC-13 cells, comparing its expression with that of a pDL-N construct containing the 5′-UTR of ODC, the mRNA of which is known to be eIF4B-dependent for translation [26], and with the pDL-N control. The relative reporter activities of the constructs containing the Lamγ2 or ODC 5′-UTR were reduced ∼50% by the RSK inhibitor BI-D1870 relative to controls (Fig. 3B). Consistent with our Western blot analyses of neoplastic epithelial cells above, the mTOR/S6K1 inhibitor Ku-0063794 had no inhibitory effect on expression of the Lamγ2 or ODC 5′-UTR reporter constructs (Fig. 3B).

### eIF4B Laminin γ2 translation requires active eIF4B because of the 5′-UTR sequence of its mRNA

EIF4B interacts with and potentiates the activity of eIF4A, an RNA helicase that unwinds secondary structures in the 5′-UTRs of certain mRNAs to expose the AUG translation initiation site [22], [24], [45]. We previously noted that the 5′-UTR of Lamγ2 mRNA is long (314 bases), GC-rich (63.4%), and energy favored for stable stem-loop formation (energy/base −0.46 kcal/mol) [21], similar to these features of the 5′-UTRs of MYC and ODC. We therefore sought to test the hypothesis that Lamγ2 expression is dependent upon eIF4B by testing the effects of shRNA-mediated eIF4B knockdown.

**Figure 4:**
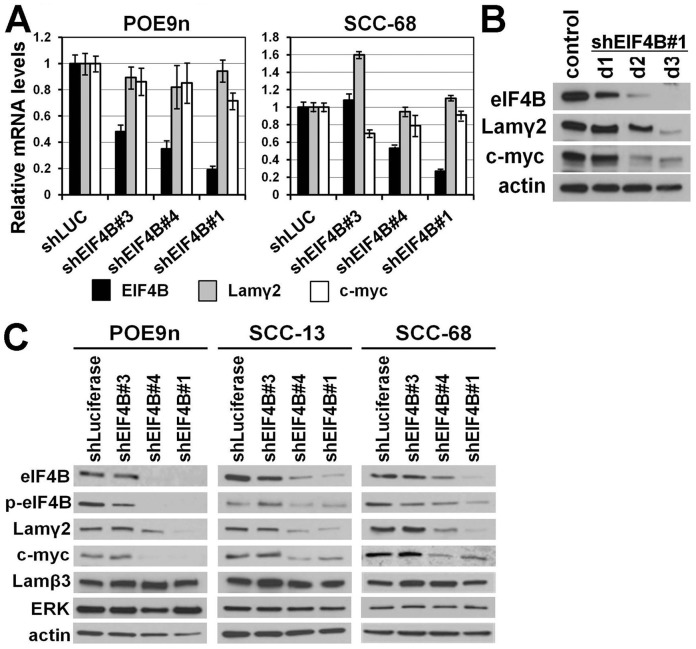
The eIF4B requirement for Lamγ2 and MYC protein expression demonstrated by shRNA knockdown. **A**) Effect of eIF4B knockdown on eIF4B, Lamγ2, and MYC mRNA levels. RNA from POE9n and SCC-68 cells transduced to express shRNAs targeting eIF4B or Luciferase was analyzed by qPCR for eIF4B, Lamγ2, and MYC transcripts. Results of each cell line was internally normalized to GAPDH mRNA levels and expressed relative to that of cells transduced to express shLuciferase, set at an arbitrary value of 1. **B**) Western blot analysis of the time-course of eIF4B protein reduction in SCC cells transduced to express shRNA.eIF4B#1 and examined 1, 2, and 3 d after transduction. **C**) Western blot analysis of the premalignant cell line (POE9n) and two SCC lines (SCC-13, SCC-68) transduced with three shRNA vectors targeting EIF4B and a control shRNA vector targeting luciferase.

We transduced POE9n and SCC-68 cells to express either a control shLuciferase or three different shEIF4Bs and 4d later determined the effects on eIF4B mRNA levels (Fig. 4A). eIF4B shRNA clones #1 and #4 resulted in the greatest eIF4B mRNA knockdown, from 50–80%. As expected, the levels of Lamγ2 and MYC mRNAs were not affected (Fig. 4A), consistent with eIF4B regulating Lamγ2 and MYC expression at the level of translation and not transcription. To determine the ability of sh.eIF4B to reduce eIF4B protein levels, its time-course, and its consequence for Lamγ2 and MYC protein expression, we transduced SCC cells with the most potent eIF4B shRNA (clone #1) and analyzed cells 1, 2 and 3 d later by Western blotting. EIF4B protein levels were substantially reduced by 2 d and reduced further 3 d after transduction (Fig. 4B). Levels of the short half-life MYC protein were greatly reduced by 2 d, whereas Lamγ2 levels were substantially reduced by 3 d after transduction (Fig. 4B).

We next engineered premalignant (POE9n) keratinocytes and SCC cells (SCC-13 and SCC-68) to express eIF4B shRNA and analyzed them by Western blotting at 4 d post-transduction. As expected from the qPCR and Western blot results (Fig. 4A,B), both eIF4B shRNA clones #1 and #4 yielded highly efficient reduction of eIF4B protein and consequently of p-eIF4B levels (Fig. 4C). Clones #1 and #4 also reduced MYC and Lamγ2 protein levels, firmly demonstrating that eIF4B is essential for Lamγ2 and MYC protein expression. Western blotting for Lamβ3 and ERK showed eIF4B knockdown had specific effects on Lamγ2 and MYC and did not result from general translation inhibition (Fig. 4C).

## Discussion

Understanding the molecular mechanisms underlying neoplasia-associated biomarkers, such as Lamγ2 and p-S6(235), is very important for achieving earlier and more reliable detection of premalignancy as well as identifying targets for small molecule anti-cancer therapeutics. Here we have elucidated the mechanism underlying MAPK-dependent Lamγ2 overexpression, a biomarker that appears in regions of many dysplastic epithelial lesions and persists in invasive SCC. Interestingly, expression of the growth-promoting transcription factor and oncogene MYC is regulated in parallel with Lamγ2 by the RSK/eIF4B-dependent mechanism that enables translation of mRNAs with long, highly structured 5′UTRs.

The premalignant dysplastic cell line POE9n and the SCC lines SCC-68 and SCC-13 we studied here do not contain integrated HPV genomic DNA. An alternate pathway of transformation in stratified squamous epithelia involves HPV16/18 infection, genomic integration, and consequent expression of the E6 and E7 viral oncoproteins as an early step [46]. Although rare in oral cavity dysplasia and SCC, high risk HPV accounts for a large proportion of vulvar dysplasias and SCC and we have found that basal p-S6(235) and Lamγ2 immunostaining in such lesions is similar to that of non HPV-related oral and vulvar lesions [21], [43]. Our present study has found that a function of HPV16E6 separate from its ability to render cells p53-deficient activates the MAPK/RSK/eIF4B pathway to cause Lamγ2 and MYC overexpression. Our study confirmed an earlier report [44] that described E6 activation of mTOR/S6K1, but we found that activation of this pathway is not essential for Lamγ2 and MYC overexpression.

We noted previously [21] that the Lamγ2 mRNA contains upstream of the translation initiation site a 314 bp sequence predicted to form a highly stem-looped structure that would require unraveling by the eIF4B-dependent eIF4A helicase. Here we have found by sh.eIF4B knockdown experiments in SCC cells that this translation factor is essential for Lamγ2 protein synthesis and also (as expected from previous studies [26]) for MYC protein synthesis. Furthermore, our reporter analysis showed that the 5′-UTR sequence of Lamγ2 confers upon its mRNA RSK-dependence for translation into protein.

The experiments described here have identified phosphorylation of ribosomal protein S6 at its S240 and S235 residues as specific readouts of mTOR hyperactivity vs. combined mTOR and MAPK hyperactivity, respectively. Early test tube studies using purified rat S6K1 and ribosomes concluded that S6K1 can phosphorylate both sites on S6 [47]. Subsequent studies in cells [48], [49] found that RSK is primarily responsible for S6(S235) phosphorylation and perusal of the data in these reports suggested to us that S235 phosphorylation is dependent upon or greatly enhanced by prior S240 phosphorylation by S6K1. Our results establish clearly that in premalignant keratinocytes and SCC cells S6(S235) phosphorylation is accomplished exclusively by RSK and requires prior phosphorylation of S240 by S6K1. *In vivo*, activation of both pathways, detectable immunohistochemically with antibodies specific for these two S6 phosphorylation sites, appears to occur at the same time during neoplastic progression, consistent with a single event being responsible for activating both pathways. The common step could be mitogen binding to and activation of EGFR, which activates the MAPK and PI3K and pathways [50]. On the other hand, our study found that a PI3K inhibitor did not reduce S6(S240) phosphorylation in culture, even while greatly reducing AKT phosphorylation. Thus, mTOR/S6K1 activity is independent of PI3K in our culture system, as expected for cells that are not subject to nutrient or growth factor limitation [51]. The use of p-S6(S240) and p-S6(S235) as immunohistochemical biomarkers for mTOR/S6K1 and MAPK/RSK pathway activation has potential application in analyzing epithelial lesions for potential premalignant change. These antigens are abundant and well-preserved in the basal, proliferative cell populations of dysplasias and tumors, unlike other phosphoproteins such as p-eIF4B in the MAPK pathway and p-eIF4E in the mTOR pathway [21].

Lamγ2 overexpression is a rather early event in dysplastic, noninvasive lesions that may progress to SCC [19]. As shown here and in our recent studies, Lamγ2 overexpression always occurs *in vivo* in cells that have increased levels of p-S6(S235) and p-S6(S240) [21], [43]. p-S6 immunostaining in the basal cell layer of dysplasias also shows promise as a potential biomarker heralding more probable future progression to invasive SCC. Some p-S6 positive regions of dysplastic lesions do not overexpress Lamγ2. Concomitant Lamγ2 staining may indicate higher and more sustained MAPK pathway hyperactivity, which may signify a greater risk of progression. Prospective studies of serial biopsies taken from the same patient, immunostaining for p-S6(S235) and Lamγ2, will be necessary to test the hypothesis that one or both of these biomarkers of MAPK/RSK activation predicts future progression to invasive SCC. Our results also support studies to determine the effectiveness of small molecule inhibitors of eIF4B, or of the eIF4A helicase it activates, to treat SCCs or to shrink or ablate potentially premalignant lesions, especially considering that translation of the growth-inducing transcription factor MYC is also eIF4B/eIF4A-dependent. Several natural and synthetic eIF4A inhibitors have been identified and are being studied for their specificity, toxicity, and potential activity as anti-cancer agents [52]-[54].

## References

[1] Siegel R, Naishadham D, Jemal A (2012) Cancer statistics, 2012. CA Cancer J Clin 62: 10–29. 22237781 10.3322/caac.20138

[2] Leemans CR, Braakhuis BJ, Brakenhoff RH (2011) The molecular biology of head and neck cancer. Nat Rev Cancer 11: 9–22. 21160525 10.1038/nrc2982

[3] Stransky N, Egloff AM, Tward AD, Kostic AD, Cibulskis K, et al (2011) The mutational landscape of head and neck squamous cell carcinoma. Science 333: 1157–1160. 21798893 10.1126/science.1208130 PMC3415217

[4] Klein JD, Grandis JR (2010) The molecular pathogenesis of head and neck cancer. Cancer Biol Ther 9: 1–7. 20038820 10.4161/cbt.9.1.10905 PMC3138532

[5] Molinolo AA, Amornphimoltham P, Squarize CH, Castilho RM, Patel V, et al (2009) Dysregulated molecular networks in head and neck carcinogenesis. Oral Oncol 45: 324–334. 18805044 10.1016/j.oraloncology.2008.07.011 PMC2743485

[6] Hobbs CG, Sterne JA, Bailey M, Heyderman RS, Birchall MA, et al (2006) Human papillomavirus and head and neck cancer: a systematic review and meta-analysis. Clin Otolaryngol 31: 259–266. 16911640 10.1111/j.1749-4486.2006.01246.x

[7] Lingen MW, Xiao W, Schmidt A, Jiang B, Pickard R, et al.. (2012) Low etiologic fraction for high-risk human papillomavirus in oral cavity squamous cell carcinomas. Oral Oncol. 10.1016/j.oraloncology.2012.07.002 22841678

[8] Syrjanen S (2005) Human papillomavirus (HPV) in head and neck cancer. J Clin Virol 32 Suppl 1S59–66. 15753013 10.1016/j.jcv.2004.11.017

[9] Argiris A, Karamouzis MV, Raben D, Ferris RL (2008) Head and neck cancer. Lancet 371: 1695–1709. 18486742 10.1016/S0140-6736(08)60728-X PMC7720415

[10] Chen LF, Cohen EE, Grandis JR (2010) New strategies in head and neck cancer: understanding resistance to epidermal growth factor receptor inhibitors. Clin Cancer Res 16: 2489–2495. 20406834 10.1158/1078-0432.CCR-09-2318 PMC2887084

[11] Chen SJ, Nakahara T, Takahara M, Kido M, Dugu L, et al (2009) Activation of the mammalian target of rapamycin signaling pathway in epidermal tumours and its correlation with cyclin-dependent kinase 2. Br J Dermatol 160: 442–445. 19016696 10.1111/j.1365-2133.2008.08903.x

[12] Napier SS, Speight PM (2008) Natural history of potentially malignant oral lesions and conditions: an overview of the literature. J Oral Pathol Med 37: 1–10. 18154571 10.1111/j.1600-0714.2007.00579.x

[13] Bremmer JF, Brakenhoff RH, Broeckaert MA, Belien JA, Leemans CR, et al (2011) Prognostic value of DNA ploidy status in patients with oral leukoplakia. Oral Oncol 47: 956–960. 21880540 10.1016/j.oraloncology.2011.07.025

[14] Holmstrup P, Vedtofte P, Reibel J, Stoltze K (2006) Long-term treatment outcome of oral premalignant lesions. Oral Oncol 42: 461–474. 16316774 10.1016/j.oraloncology.2005.08.011

[15] van der Waal I (2009) Potentially malignant disorders of the oral and oropharyngeal mucosa; terminology, classification and present concepts of management. Oral Oncol 45: 317–323. 18674954 10.1016/j.oraloncology.2008.05.016

[16] Koshikawa N, Moriyama K, Takamura H, Mizushima H, Nagashima Y, et al (1999) Overexpression of laminin γ2 chain monomer in invading gastric carcinoma cells. Cancer Res 59: 5596–5601. 10554040

[17] Ono Y, Nakanishi Y, Ino Y, Niki T, Yamada T, et al (1999) Clinocopathologic significance of laminin-5 γ2 chain expression in squamous cell carcinoma of the tongue: immunohistochemical analysis of 67 lesions. Cancer 85: 2315–2321. 10357399

[18] Pyke C, Romer J, Kallunki P, Lund LR, Ralfkiaer E, et al (1994) The γ2 chain of kalinin/laminin 5 is preferentially expressed in invading malignant cells in human cancers. Am J Pathol 145: 782–791. 7943170 PMC1887322

[19] Natarajan E, Saeb M, Crum CP, Woo SB, McKee PH, et al (2003) Co-expression of p16^INK4A^ and laminin 5 γ2 by microinvasive and superficial squamous cell carcinomas in vivo and by migrating wound and senescent keratinocytes in culture. Am J Pathol 163: 477–491. 12875969 10.1016/s0002-9440(10)63677-2 PMC1868206

[20] Nordemar S, Hogmo A, Lindholm J, Auer G, Munck-Wikland E (2003) Laminin-5 γ2: a marker to identify oral mucosal lesions at risk for tumor development? Anticancer Res 23: 4985–4989. 14981956

[21] Degen M, Natarajan E, Barron P, Widlund HR, Rheinwald JG (2012) MAPK/ERK-dependent translation factor hyperactivation and dysregulated Laminin γ2 expression in oral dysplasia and squamous cell carcinoma. Am J Pathol 180: 2462–2478. 22546478 10.1016/j.ajpath.2012.02.028 PMC3378915

[22] Lawson TG, Lee KA, Maimone MM, Abramson RD, Dever TE, et al (1989) Dissociation of double-stranded polynucleotide helical structures by eukaryotic initiation factors, as revealed by a novel assay. Biochemistry 28: 4729–4734. 2548591 10.1021/bi00437a033

[23] Rogers GW Jr, Richter NJ, Lima WF, Merrick WC (2001) Modulation of the helicase activity of eIF4A by eIF4B, eIF4H, and eIF4F. J Biol Chem 276: 30914–30922. 11418588 10.1074/jbc.M100157200

[24] Rozen F, Edery I, Meerovitch K, Dever TE, Merrick WC, et al (1990) Bidirectional RNA helicase activity of eucaryotic translation initiation factors 4A and 4F. Mol Cell Biol 10: 1134–1144. 2304461 10.1128/mcb.10.3.1134-1144.1990 PMC360981

[25] Shahbazian D, Parsyan A, Petroulakis E, Hershey J, Sonenberg N (2010) eIF4B controls survival and proliferation and is regulated by proto-oncogenic signaling pathways. Cell Cycle 9: 4106–4109. 20948310 10.4161/cc.9.20.13630 PMC3055195

[26] Shahbazian D, Parsyan A, Petroulakis E, Topisirovic I, Martineau Y, et al (2010) Control of cell survival and proliferation by mammalian eukaryotic initiation factor 4B. Mol Cell Biol 30: 1478–1485. 20086100 10.1128/MCB.01218-09 PMC2832492

[27] Dickson MA, Hahn WC, Ino Y, Ronfard V, Wu JY, et al (2000) Human keratinocytes that express hTERT and also bypass a p16^INK4A^-enforced mechanism that limits life span become immortal yet retain normal growth and differentiation characteristics. Mol Cell Biol 20: 1436–1447. 10648628 10.1128/mcb.20.4.1436-1447.2000 PMC85304

[28] Natarajan E, Omobono JD 2nd, Guo Z, Hopkinson S, Lazar AJ, et al (2006) A keratinocyte hypermotility/growth-arrest response involving Laminin 5 and p16^INK4A^ activated in wound healing and senescence. Am J Pathol 168: 1821–1837. 16723698 10.2353/ajpath.2006.051027 PMC1606631

[29] Rheinwald JG, Beckett MA (1980) Defective terminal differentiation in culture as a consistent and selectable character of malignant human keratinocytes. Cell 22: 629–632. 6160916 10.1016/0092-8674(80)90373-6

[30] Dabelsteen S, Hercule P, Barron P, Rice M, Dorsainville G, et al (2009) Epithelial cells derived from human embryonic stem cells display p16INK4A senescence, hypermotility, and differentiation properties shared by many p63+ somatic cell types. Stem Cells 27: 1388–1399. 19489101 10.1002/stem.64 PMC2733375

[31] Foster SA, Demers GW, Etscheid BG, Galloway DA (1994) The ability of human papillomavirus E6 proteins to target p53 for degradation in vivo correlates with their ability to abrogate actinomycin D-induced growth arrest. J Virol 68: 5698–5705. 8057451 10.1128/jvi.68.9.5698-5705.1994 PMC236972

[32] Mietz JA, Unger T, Huibregtse JM, Howley PM (1992) The transcriptional transactivation function of wild-type p53 is inhibited by SV40 large T-antigen and by HPV-16 E6 oncoprotein. Embo J 11: 5013–5020. 1464323 10.1002/j.1460-2075.1992.tb05608.x PMC556979

[33] DuBridge RB, Tang P, Hsia HC, Leong PM, Miller JH, et al (1987) Analysis of mutation in human cells by using an Epstein-Barr virus shuttle system. Mol Cell Biol 7: 379–387. 3031469 10.1128/mcb.7.1.379-387.1987 PMC365079

[34] Greulich H, Chen TH, Feng W, Janne PA, Alvarez JV, et al (2005) Oncogenic transformation by inhibitor-sensitive and -resistant EGFR mutants. PLoS Med 2: e313. 16187797 10.1371/journal.pmed.0020313 PMC1240052

[35] Wakeling AE, Guy SP, Woodburn JR, Ashton SE, Curry BJ, et al (2002) ZD1839 (Iressa): an orally active inhibitor of epidermal growth factor signaling with potential for cancer therapy. Cancer Res 62: 5749–5754. 12384534

[36] Sapkota GP, Cummings L, Newell FS, Armstrong C, Bain J, et al (2007) BI-D1870 is a specific inhibitor of the p90 RSK (ribosomal S6 kinase) isoforms in vitro and in vivo. Biochem J 401: 29–38. 17040210 10.1042/BJ20061088 PMC1698666

[37] Favata MF, Horiuchi KY, Manos EJ, Daulerio AJ, Stradley DA, et al (1998) Identification of a novel inhibitor of mitogen-activated protein kinase kinase. J Biol Chem 273: 18623–18632. 9660836 10.1074/jbc.273.29.18623

[38] Dowling RJ, Topisirovic I, Fonseca BD, Sonenberg N (2010) Dissecting the role of mTOR: lessons from mTOR inhibitors. Biochim Biophys Acta 1804: 433–439. 20005306 10.1016/j.bbapap.2009.12.001

[39] Garcia-Martinez JM, Moran J, Clarke RG, Gray A, Cosulich SC, et al (2009) Ku-0063794 is a specific inhibitor of the mammalian target of rapamycin (mTOR). Biochem J 421: 29–42. 19402821 10.1042/BJ20090489 PMC2708931

[40] Knight ZA, Gonzalez B, Feldman ME, Zunder ER, Goldenberg DD, et al (2006) A pharmacological map of the PI3-K family defines a role for p110α in insulin signaling. Cell 125: 733–747. 16647110 10.1016/j.cell.2006.03.035 PMC2946820

[41] Mizushima H, Koshikawa N, Moriyama K, Takamura H, Nagashima Y, et al (1998) Wide distribution of laminin-5 γ2 chain in basement membranes of various human tissues. Horm Res 50 Suppl 27–14. 9721586 10.1159/000053118

[42] Venkatesan A, Sharma R, Dasgupta A (2003) Cell cycle regulation of hepatitis C and encephalomyocarditis virus internal ribosome entry site-mediated translation in human embryonic kidney 293 cells. Virus Res 94: 85–95. 12902037 10.1016/s0168-1702(03)00136-9

[43] Pinto AP, Degen M, Barron P, Crum CP, Rheinwald JG (2013) Phosphorylated S6 as an immunohistochemical biomarker of vulvar intraepithelial neoplasia. Mod Pathol (in press). 10.1038/modpathol.2013.85 23765247

[44] Spangle JM, Munger K (2010) The human papillomavirus type 16 E6 oncoprotein activates mTORC1 signaling and increases protein synthesis. J Virol 84: 9398–9407. 20631133 10.1128/JVI.00974-10 PMC2937655

[45] Gingras AC, Raught B, Sonenberg N (1999) eIF4 initiation factors: effectors of mRNA recruitment to ribosomes and regulators of translation. Annu Rev Biochem 68: 913–963. 10872469 10.1146/annurev.biochem.68.1.913

[46] McLaughlin-Drubin ME, Munger K (2009) Oncogenic activities of human papillomaviruses. Virus Res 143: 195–208. 19540281 10.1016/j.virusres.2009.06.008 PMC2730997

[47] Ferrari S, Bandi HR, Hofsteenge J, Bussian BM, Thomas G (1991) Mitogen-activated 70K S6 kinase. Identification of in vitro 40 S ribosomal S6 phosphorylation sites. J Biol Chem 266: 22770–22775. 1939282

[48] Pende M, Um SH, Mieulet V, Sticker M, Goss VL, et al (2004) S6K1(-/-)/S6K2(-/-) mice exhibit perinatal lethality and rapamycin-sensitive 5′-terminal oligopyrimidine mRNA translation and reveal a mitogen-activated protein kinase-dependent S6 kinase pathway. Mol Cell Biol 24: 3112–3124. 15060135 10.1128/MCB.24.8.3112-3124.2004 PMC381608

[49] Roux PP, Shahbazian D, Vu H, Holz MK, Cohen MS, et al (2007) RAS/ERK signaling promotes site-specific ribosomal protein S6 phosphorylation via RSK and stimulates cap-dependent translation. J Biol Chem 282: 14056–14064. 17360704 10.1074/jbc.M700906200 PMC3618456

[50] Castellano E, Downward J (2011) RAS interaction with PI3K: more than just another effector pathway. Genes Cancer 2: 261–274. 21779497 10.1177/1947601911408079 PMC3128635

[51] Inoki K, Zhu T, Guan KL (2003) TSC2 mediates cellular energy response to control cell growth and survival. Cell 115: 577–590. 14651849 10.1016/s0092-8674(03)00929-2

[52] Bordeleau ME, Mori A, Oberer M, Lindqvist L, Chard LS, et al (2006) Functional characterization of IRESes by an inhibitor of the RNA helicase eIF4A. Nat Chem Biol 2: 213–220. 16532013 10.1038/nchembio776

[53] Cencic R, Carrier M, Galicia-Vazquez G, Bordeleau ME, Sukarieh R, et al (2009) Antitumor activity and mechanism of action of the cyclopenta[b]benzofuran, silvestrol. PLoS One 4: e5223. 19401772 10.1371/journal.pone.0005223 PMC2671147

[54] Jin C, Rajabi H, Rodrigo CM, Porco JA Jr, Kufe D (2012) Targeting the eIF4A RNA helicase blocks translation of the MUC1-C oncoprotein. Oncogene 32: 2179–2188. 22689062 10.1038/onc.2012.236 PMC3443512

